# Pulmonary resection and systemic lymph node dissection in a patient with breast cancer who had a 33-year disease-free interval

**DOI:** 10.1186/s12957-015-0565-y

**Published:** 2015-04-16

**Authors:** Degang Yin, Guofei Zhang, Lufeng Zhao, Ying Chai

**Affiliations:** Department of Thoracic Surgery, The Second Affiliated Hospital, College of Medicine, Zhejiang University, # 88 Jiefang Road, Hangzhou, 310009 China

**Keywords:** Breast cancer, Pulmonary metastasis, Mediastinal lymph node metastasis, Disease-free interval

## Abstract

**Objective:**

Breast cancer metastasis to the lung is common. The resection of lung metastases in patients with breast cancer has been controversial. Here, we present a very rare case of pulmonary and mediastinal lymph node metastases in a patient with breast cancer who had a disease-free interval (DFI) of more than 33 years.

**Methods:**

An involved lobectomy and systematic mediastinal lymph node dissection were performed.

**Results:**

The histological examination confirmed pulmonary metastasis from the breast cancer associated with mediastinal lymph nodes metastasis.

**Conclusions:**

To our knowledge, this is the first case reported of a patient with a 33-year DFI after a radical mastectomy for breast cancer who presented with pulmonary metastasis with mediastinal lymph node involvement. This case indicates that a long-term follow-up of breast cancer patients is necessary. Systematic mediastinal lymph node dissection should be considered as a prognostic study during pulmonary metastasectomy for breast cancer.

## Background

The lung is a common location for the occurrence of malignant metastases. There is a high likelihood of pulmonary metastasis developing in patients with breast cancer [1]. Patients with isolated lung metastases are rare; usually, local or diffuse lymphangitis with single or multiple lung metastases are found [2]. Metastatic breast cancer has been defined as a systemic disease and a terminal cancer. The resection of lung metastases in patients with breast cancer has been controversial [3-7]. However, discussions on the criteria for pulmonary metastasectomy seem to have currently reached a consensus [8]: control of the primary malignancy, complete resectability of the pulmonary lesions, and the presence of no other - extrapulmonary - metastasis.

A disease-free interval (DFI) of greater than 10 years has been reported only occasionally [7,9-11]. To our knowledge, the longest DFI reported previously was 27 years [10]. Recently, we diagnosed and treated a very rare case of pulmonary and mediastinal metastases in a patient with breast cancer who had a 33-year DFI.

## Case presentation

A 61-year-old woman was admitted to our hospital with chest pain for 2 months. She underwent a radical mastectomy for right breast cancer 33 years earlier.

**Figure 1:**
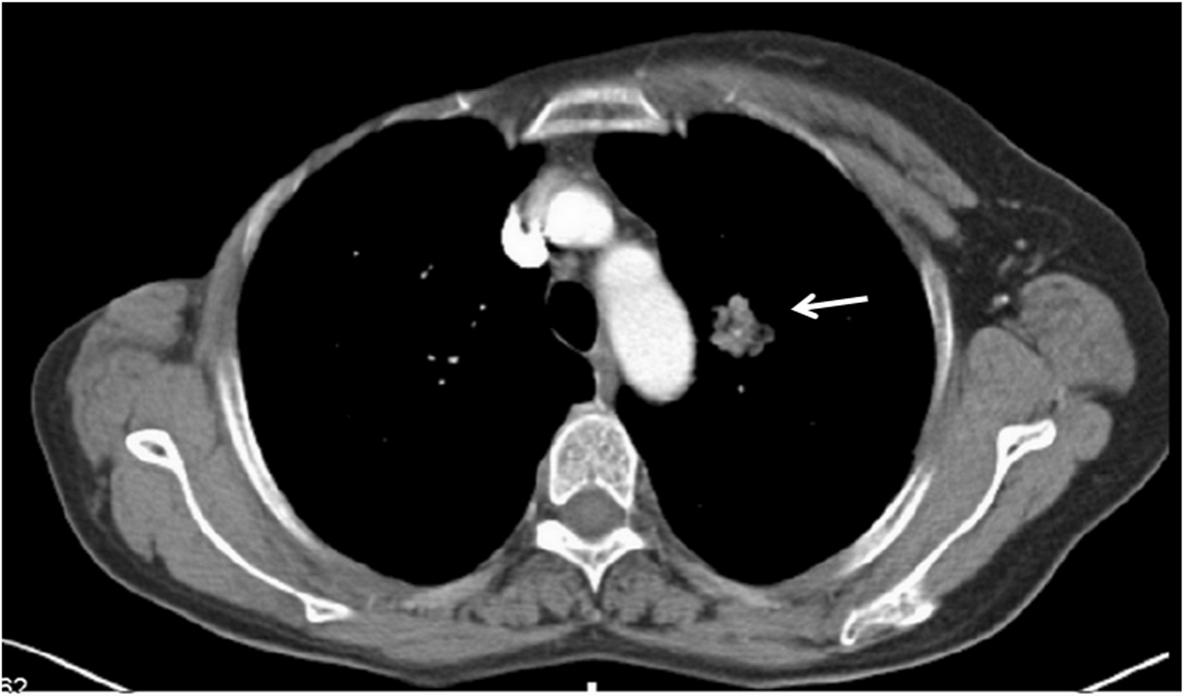
Enhancement computed tomography scan of chest reveals a 2.3 × 2.3 cm lobulated tumor in the left lung.

Chest computed tomography revealed an approximately 2.3 × 2.3 cm heterogeneous mass with a burr in the left upper lobe of the lung, but no obvious lymph node swelling was observed in the mediastinum (Figure 1). A preliminary diagnosis of primary lung cancer was made, followed by plans for surgery.

A left thoracotomy was performed, and the tumor was discovered in the upper lobe of the left lung, which appeared as a gray fish-like tissue. The hilar, mediastinal, and bronchial nodes nearby were palpable, and there were more swollen lymph nodes. A segmentectomy of the left upper lobe of the lung was performed for tissue confirmation. A frozen section removed from the mass revealed a malignant epithelial tumor. Then, a left upper lobectomy and systemic lymph node dissection were performed.

**Figure 2:**
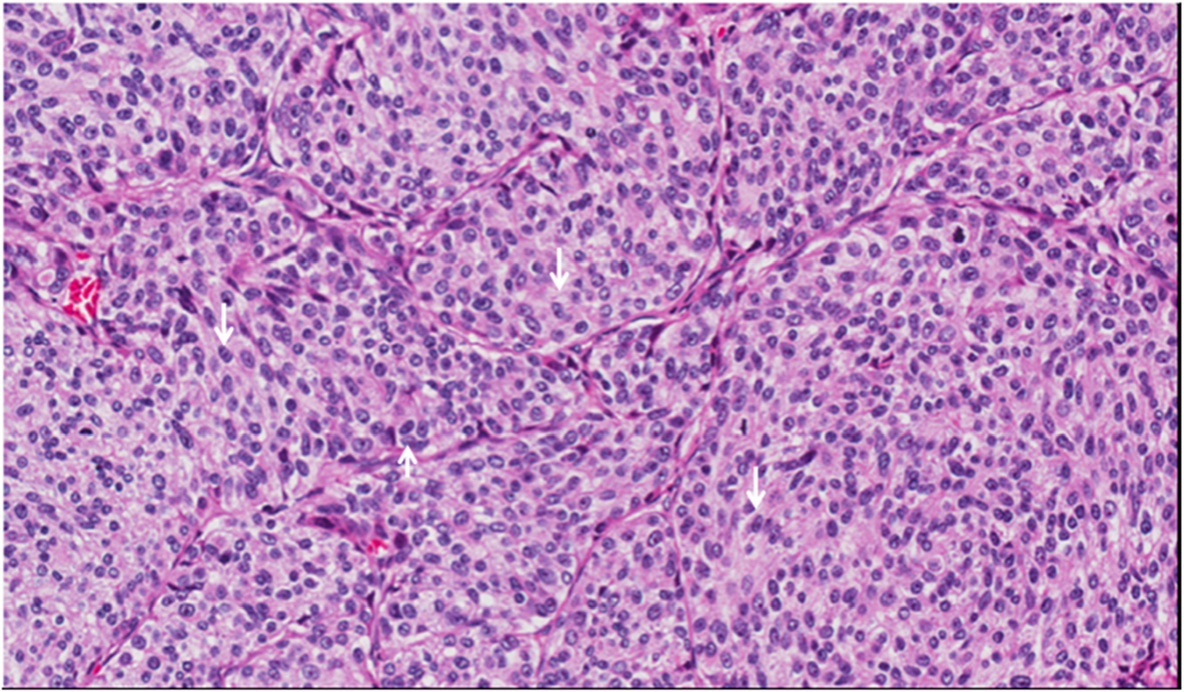
Lung postoperative pathology (H & E staining, ×200): metastatic breast ductal carcinoma.

**Figure 3:**
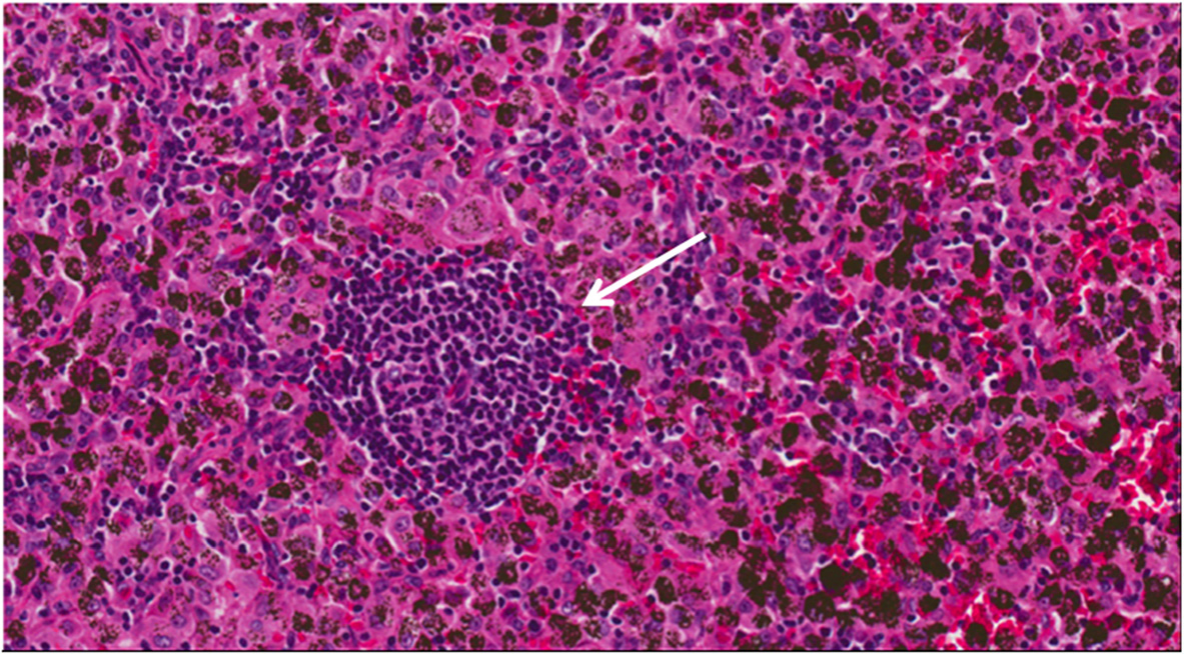
Mediastinal lymph node postoperative pathology (H & E staining, ×200): metastatic breast ductal carcinoma.

**Figure 4:**
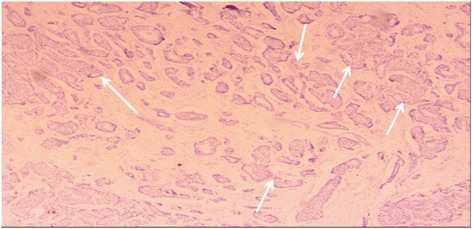
Breast cancer postoperative pathology (H & E staining, ×40): small portion of visible tumors are arranged in the duct gland (infiltrating ductal carcinoma).

However, the final pathology confirmed breast ductal cancer metastasis (Figure 2). The largest diameter of the tumor was 2 cm, and the left lower paratracheal and subaortic lymph nodes were positive (Figure 3). The immunohistochemical results were as follows: estrogen receptor + + +, progesterone receptor +, human epidermal growth factor receptor 2 (HER2) + +, and Ki67 (20 to 30%). Overexpression and amplification of HER2 were detected by FISH. Moreover, the pathological examination showed similar results to those observed in the specimen 33 years earlier (Figure 4). As a result, a metastatic ductal carcinoma from the breast was diagnosed. Additionally, the left lower paratracheal and subaortic lymph nodes were positive (Figure 3), but the other lymph nodes were negative.

The patient had an uneventful postoperative recovery. On the ninth postoperative day, she was discharged and sent home with oral anastrozole 1 mg/day. The 1-year follow-up was uneventful.

## Discussion

Breast cancer has a tendency to relapse after a long DFI. In a literature search of English and Japanese publications, the longest DFI reported previously was 27 years [10]. We found no case with a DFI greater than 30 years. In this case, pulmonary and associated mediastinal lymph node metastases occurred in a patient with breast cancer and a DFI of 33.6 years (403 months), indicating that a long-term follow-up is necessary.

Advanced breast cancer often infiltrates the lungs and pleurae by direct invasion, lymphogenic dissemination, or hematogenic dissemination [2]. What is the mechanism by which breast cancer cells lie dormant over very long durations in the lung? It may be related to the body’s immunity and the dormancy and activation mechanisms of breast cancer cells. Tumor dormancy refers to the primary tumor after radical excision; microscopic tumors or trace tumor cells exist for a certain period of time, with no proliferation or growth. However, these ‘cancer stem cells’ still have the potential for proliferation, which can occur several years or even decades later, leading to tumor recurrence or distant metastasis [12]. Thus, immunotherapy or endocrinotherapy is recommended after surgery or radiotherapy and chemotherapy.

Breast cancer metastasis to the lung is common. Discussions concerning lung metastasectomy have been controversial [3]. Today, there is a consensus on the indications for pulmonary metastatic tumor resection [8,13,14]: (1) the primary malignant tumor has been ‘cured,’ (2) imaging suggests that all metastases can be removed, (3) there is no other - extrapulmonary - metastasis, (4) cardiopulmonary function and the general conditions of the patient are appropriate for pulmonary metastatic resection, (5) the patient has no serious pleural adhesion, and the mediastinum has no obvious swelling or calcified lymph nodes, and (6) there is no other more effective treatment. An unresectable primary tumor and predicted incomplete metastasectomy are absolute contraindications to a lung metastasectomy [14]. Literature reports confirm that most patients with pulmonary metastases who could not undergo a complete resection had a poor prognosis [15-17].

The general principle of surgical treatment of pulmonary metastases is complete resection of the metastases and removal of as little healthy lung tissue as possible, which makes repeated pulmonary metastasectomies still acceptable. The surgical options are determined by the size, position, and number of metastases [8,13,14]. If metastases are located within a single lung segment, a segmentectomy is appropriate. If multiple metastases are located in the same lobe, or metastases are located near the hilum or hilar lymph nodes, patients can undergo a lobectomy. If metastases located in the hilar or metastases are larger and infringe upon two lobes, two lobectomies or a pneumonectomy may be performed [8,13,14]. Because pulmonary metastases tend to be located peripherally, a wedge resection is a common procedure [8]. However, it is difficult clinically to distinguish between primary lung cancer and pulmonary metastasis [7]. Thus, a lobectomy and systematic mediastinal lymph node dissection were performed in this case. However, the finally pathology confirmed ductal breast cancer metastasis.

Although many consider it unnecessary to perform systematic mediastinal lymph node dissection, its potential therapeutic effect remains unknown. Thus, whether a systematic mediastinal lymph node dissection should be performed is an interesting topic. In this case, systematic mediastinal lymph node dissection was performed, which was essentially ‘accidental,’ but so far the effect has been very good. If a systematic mediastinal lymph node dissection had not been performed, which resulted in tumor residue, a subsequently poor response to adjuvant chemotherapy and hormone therapy could indicate a poor prognosis. Thus, we suggest that systematic mediastinal lymph node dissection be considered to determine the prognosis during solitary pulmonary metastasectomy in patients with breast cancer. Further studies using a prospective design are warranted to evaluate the relationship between systematic mediastinal lymph node dissection and prognosis.

## Conclusions

To our knowledge, this is the first case reported of a patient with a 33-year DFI after a radical mastectomy for breast cancer who presented with pulmonary metastasis with mediastinal lymph node involvement. This case indicates that a long-term follow-up of breast cancer patients is necessary. Systematic mediastinal lymph node dissection should be considered as a prognostic study during pulmonary metastasectomy for breast cancer.

## Consent

Written informed consent was obtained from the patient for publication of this case report and any accompanying images. A copy of the written consent is available for review by the Editor-in-Chief of this journal.
